# Strong Adherence to Cardiac Rehabilitation Program Improves Exercise Tolerance in Outpatients With Cardiovascular Disease

**DOI:** 10.14740/cr2213

**Published:** 2026-06-05

**Authors:** Kakeru Bando, Yasunori Suematsu, Takuro Matsuda, Yuiko Yano, Kai Tsukahara, Takashi Maruo, Hiroyuki Fukuda, Chie Matsushita, Etsumi Nakamura, Reiko Teshima, Hinata Oda, Kanta Fujimi, Shin-ichiro Miura

**Affiliations:** a Department of Cardiology, Shin-Yukuhashi Hospital, Fukuoka, Japan; b Department of Cardiology, Fukuoka University Hospital, Fukuoka, Japan; c Department of Rehabilitation, Fukuoka University Hospital, Fukuoka, Japan; d Miyase Clinic, Fukuoka, Japan; e Hinoki Clinic, Fukuoka, Japan; f Department of Nursing, Fukuoka University Hospital, Fukuoka, Japan; g Department of Nutrition, Fukuoka University Hospital, Fukuoka, Japan; h Fukuoka University School of Medicine, Fukuoka, Japan; i Department of Cardiology, School of Medicine, Fukuoka University, Fukuoka, Japan

**Keywords:** Cardiac rehabilitation, Frequency, Exercise tolerance

## Abstract

**Background:**

Exercise-based cardiac rehabilitation (CR) programs are recommended in outpatients at least three times a week, but high-frequency visits can be difficult due to patients’ work schedules. The association between the frequency of exercise-based CR and the increase in exercise tolerance is not well known. We evaluated the effect of CR once a week or more for improving exercise tolerance.

**Methods:**

This is a retrospective observational study. From February 2011 to January 2020, 110 outpatients who participated in CR and performed a cardiopulmonary exercise test (CPET) two times (initially and within 6 months) were registered. We divided the patients into those who participated in CR once a week or more (n = 75) and less than once a week (n = 35) and compared the changes in the results of CPET.

**Results:**

Overall, the median patient age was 68.5 (59–74) years, 69.1% were males, and the body mass index was 23.8 (20.2–27.4) kg/m^2^. The average frequency of CR was 1.54 (0.93–1.89) times per week. While patients who participated in CR once a week or more showed improvements in oxygen uptake, ventilatory equivalent, ventilatory efficiency, and oxygen uptake per heart rate, those who participated in CR less than once a week showed only small improvements. The ratio of oxygen uptake to work rate in patients who participated in CR once a week or more also improved significantly.

**Conclusions:**

Patients who participated in a CR program once a week or more showed improved exercise tolerance.

## Introduction

Cardiac rehabilitation (CR) refers to a long-term, multifaceted, comprehensive program designed to optimize a cardiac patient’s physical, psychological, social, and vocational status, in addition to stabilizing, slowing, or even reversing the progression of the underlying atherosclerotic or heart failure processes, thereby reducing recurrence, rehospitalization, and mortality, and enabling patients to live comfortably and actively [1].

CR has been shown to improve the prognosis for patients with cardiovascular diseases [2, 3]. Exercise tolerance is associated with the patient’s quality of life and activities of daily living, and exercise-based CR has been shown to improve exercise tolerance [4, 5]. The cardiopulmonary exercise test (CPET) is used to study exercise tolerance by using expiratory gas analysis [6, 7]. Oxygen uptake during peak and anaerobic threshold periods reflects exercise tolerance. In addition to oxygen uptake, CPET is able to measure cardiac function, ventilation volume, and ventilation efficiency [6]. However, the association between the frequency of exercise-based CR and the increase in exercise tolerance is not well known.

CR is a requisite component of care for patients with heart failure and ischemic heart disease, and is a class A recommendation in international guidelines [8–10]. The guideline of American Heart Association and European Society of Cardiology recommends 3–5 days/week exercise [11]. The guideline from European Society of Cardiology recommends 150–300 min a week of moderate intensity exercise [12]. The Japanese cardiac rehabilitation guideline also recommend that CR should be performed as often as the number of times in the exercise prescription (e.g., five times/week) in some combination of supervised outpatient CR and home-based exercise training [1]. The frequency of outpatient exercise-based CR is affected by the patient’s motivation, employment/family support, financial status, and ease of access, but it is desirable for patients to participate in outpatient exercise-based CR three times each week if possible [1]. However, frequent hospital visits are difficult for patients due to lifestyle issues. In this study, we evaluated the effect of CR once a week or more on exercise tolerance, compared to CR at less than once a week.

## Materials and Methods

### Study population and protocol

**Figure 1:**
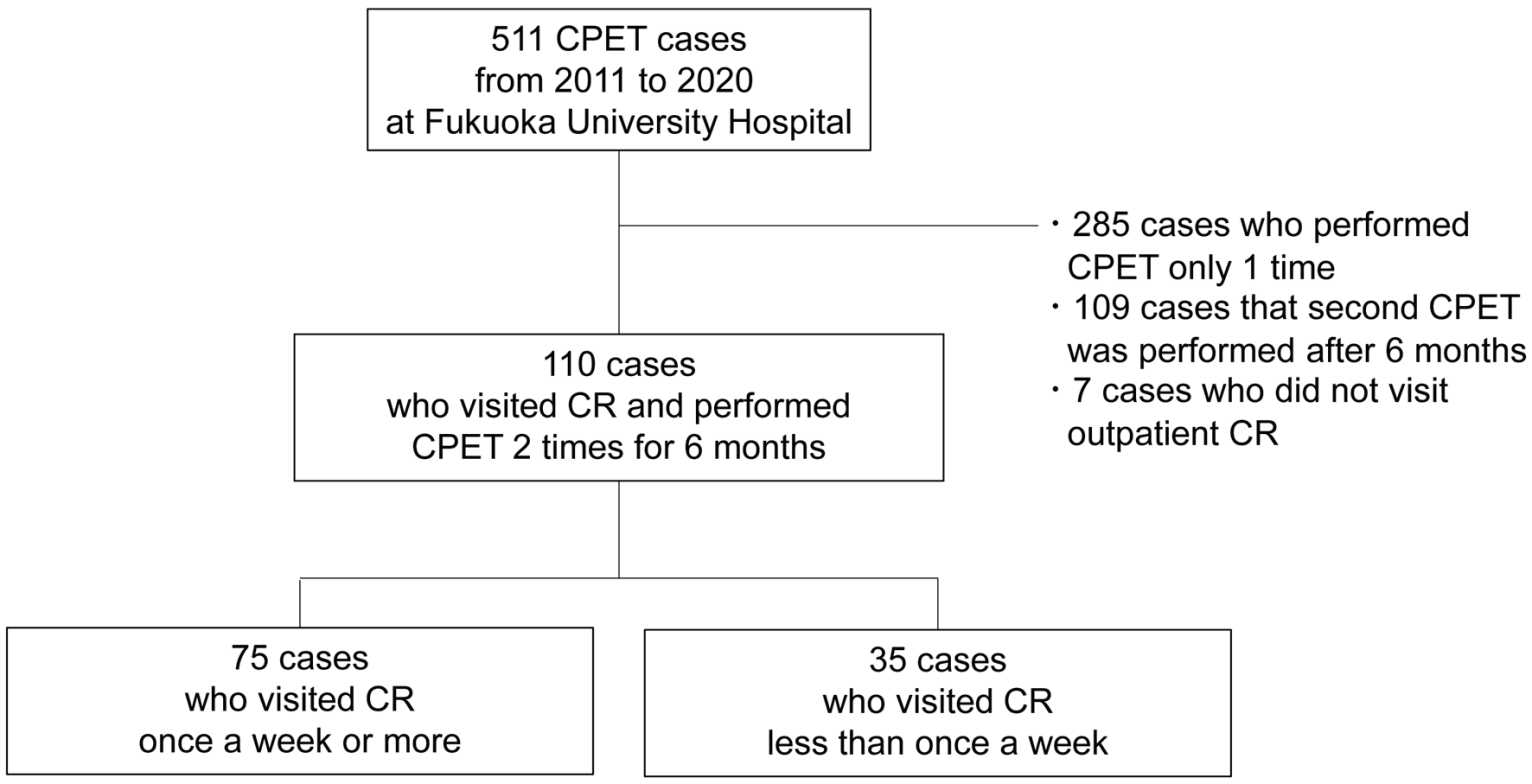
Study flow diagram. CPET: cardiopulmonary exercise test; CR: cardiac rehabilitation.

This single-center retrospective observational study evaluated the effect of CR once a week or more on exercise tolerance. We recommended three times CR sessions per week for all patients; however, the frequency to CR was decided by the patients. CR program includes medical assessment, prescribed exercise training, patient education, counseling, and optimal medical therapy by a multi-disciplinary team, followed by the guideline [1]. Exercise training was performed 30 min moderate intensity aerobic exercise and 20 min group bodyweight resistance exercise. Medical assessment, patient education, and dietary guidance was performed by on-site physician, nurse, or registered dietitian. Psychological counseling was performed by clinical psychologist or psychiatrist, if the patient needs it. Between February 2011 and January 2020, 511 consecutive CPET cases were registered at Fukuoka University Hospital [13, 14]. In this study, we included 110 outpatients who underwent CPET two times: initially and within 6 months. We excluded 285 cases in which CPET was performed only once, 109 cases in which the second evaluation was performed after 6 months, and seven cases that did not participate in outpatient CR. The study flowchart is shown in Figure 1. We divided the patients into those who participated in CR once a week or more (n = 75) and those who participated in CR less than once a week (n = 35) and compared the changes in the CPET results. The average duration of CR in CR less than once a week group was 109 ± 35 days and the average period of CR in CR once a week or more group was 105 ± 26 days. There was no significant difference. This study was approved by the ethics committee of Fukuoka University (U20-10-004), and was performed in accordance with the Declaration of Helsinki and the ethical standards of the Independent Review Board of Fukuoka University.

### CPET

CPET was performed using a ramp protocol on a cycle ergometer (Strength Ergo 8, Fukuda Denshi, Tokyo, Japan) with a 4-min rest period and a 4-min warm-up period. In warmup period, 98 cases (89.1%), four cases (3.6%), and eight cases (7.3%) were performed by 10, 20, 0 watts, respectively. In ramp period, all patients were performed by 10 watt/min increase protocol. The gas analyzer used was a CPEX1 (Inter Riha, Tokyo, Japan). A symptom-limited exercise test was then performed. The anaerobic threshold (AT) was considered to occur at a disproportionate increase in carbon dioxide output (VCO_2_) against oxygen uptake (VO_2_) (V-slope method) [7]. We analyzed heart rate (HR), systolic blood pressure, diastolic blood pressure, VO_2_, VCO_2_, respiratory exchange ratio (RER), metabolic equivalent (MET), VO_2_ per body weight (VO_2_/wt), VO_2_/HR, work rate, ventilation equivalent (VE), tidal volume, respiratory rate (RR), end-tidal oxygen (ETO_2_), end-tidal carbon dioxide (ETCO_2_), VE/VO_2_, and VE/VCO_2_, at rest, warmup, 1 min before AT, AT, and peak periods. The VE/VCO_2_ slope, minimum VE/VCO_2_, the oxygen-uptake efficiency slope (OUES), and the ratio of the increase in oxygen uptake to the increase in work rate (ΔVO_2_/ΔWR) were also evaluated.

### Patient backgrounds and the results of examinations

Age, sex, body weight, body mass index, history or presence of smoking, hypertension, diabetes mellitus, dyslipidemia, chronic kidney disease, and chronic obstructive pulmonary disease were investigated. Ischemic heart disease, chronic heart failure, and macrovascular disease were investigated as underlying cardiovascular diseases. Underlying cardiovascular disease represents the indication to CR. With regard to treatment, medications included renin-angiotensin-aldosterone system inhibitors, calcium channel blockers, diuretics, beta-blockers, mineral corticoid antagonists, and statin; percutaneous coronary intervention, coronary artery bypass grafting, cardiac valvular surgery, and implantation of an implantable cardioverter defibrillator or cardiac resynchronization therapy were investigated. The results of echocardiography and plasma total cholesterol, triglyceride, low-density lipoprotein cholesterol, high-density lipoprotein cholesterol, and brain natriuretic peptide (BNP) levels were obtained if the examinations were performed within 3 months from the day of CPET.

### Statistical analysis

The SAS Software Package (Version 9.4, SAS Institute Inc., Cary, NC, USA) was used for analyses at Fukuoka University (Fukuoka, Japan). Continuous variables with a normal distribution are expressed as the mean ± standard deviation, and differences between the groups were compared using an unpaired *t*-test. Continuous variables with a non-normal distribution are presented as the median and interquartile range, and the differences between the groups were compared using the Mann-Whitney U test. Categorical variables are presented as numbers (%), and the differences in categorical variables between the groups were compared using a Chi-square analysis. To analyze changes in CPET results, a paired a *t*-test was performed for continuous variables with a normal distribution and a Wilcoxon signed-rank test was performed for continuous variables with a non-normal distribution. Delta changes of individual parameters in CPET from the initial test to the second test between two groups were analyzed by a paired design analysis of variance. All probability values were two-tailed, and P-values < 0.05 were considered statistically significant.

## Results

### Patient characteristics at baseline in all patients and the two groups

The patient characteristics at baseline are shown in Table 1. The frequency of CR between the two CPETs was 1.54 (0.93–1.89) times per week. Only two of the patients in this study participated in CR more than three times per week. The average duration was 106 ± 29 days. Overall, the median patient age was 68.5 (59–74) years, 69.1% were males, and the body mass index was 23.8 ± 3.6 kg/m^2^. There were no significant differences in comorbidities between the groups. With regard to treatment, the use of diuretics was significantly low in patients who participated in CR once a week or more. There were no significant differences in other medications or treatments. There were also no significant differences in the serum level of BNP, left ventricular ejection fraction, or the tricuspid regurgitation pressure gradient between the groups.

**Table 1:** Patient Characteristics in All Patients and the Groups That Visited CR Less Than Once a Week Group and Once a Week More Group

Variables	All cases		CR less than once a week		CR once a week more		P value
	Missing	n = 110	Missing	n = 35	Missing	n = 75	
Frequency of CR, /week		1.54 (0.93-1.89)		0.79 (0.53–0.92)		1.77 (1.53–2.20)	< 0.001
Duration of CR, days		106 ± 29		108 ± 35		105 ± 26	0.52
Age, years		68.5 (59–74)		67 (51–74)		69 (64–74)	0.28
Male, n (%)		76 (69.1)		24 (68.6)		52 (69.3)	0.94
BMI, kg/m^2^		23.8 ± 3.6		24.0 ± 3.8		23.7 ± 3.6	0.68
Smoking, n (%)		59 (57.8)		23 (67.6)		36 (52.9)	0.16
Underlying disease							
HT, n (%)	1	74 (67.9)	1	24 (70.6)		50 (66.7)	0.68
DM, n (%)	1	34 (31.2)	1	11 (32.4)		23 (30.7)	0.86
DLP, n (%)	1	73 (67.0)	1	23 (67.6)		50 (66.7)	0.92
CKD, n (%)	1	52 (47.7)	1	16 (47.1)		36 (48.0)	0.93
COPD, n (%)	1	1 (0.9)	1	0 (0)		1 (1.3)	0.50
Underlying CVD							
IHD, n (%)		57 (51.8)		21 (60.0)		36 (48.0)	0.24
HF, n (%)	1	51 (46.8)		20 (58.8)	1	31 (41.3)	0.09
MVD, n (%)	1	9 (8.3)		1 (2.9)	1	8 (10.7)	0.17
Medication							
RAAS, n (%)	21	63 (70.8)	6	22 (75.9)	15	41 (68.3)	0.46
CCB, n (%)	21	39 (43.8)	6	10 (34.5)	15	29 (48.3)	0.22
Diuretics, n (%)	21	33 (37.1)	6	16 (55.2)	15	17 (28.3)	0.01
BB, n (%)	21	58 (65.2)	6	23 (79.3)	15	35 (58.3)	0.18
MRA, n (%)	21	26 (29.2)	6	12 (41.4)	15	14 (23.3)	0.08
Statin, n (%)	12	62 (63.3)	3	20 (62.5)	9	42 (63.6)	0.91
PCI, n (%)	1	41 (37.6)		16 (47.1)	1	25 (33.3)	0.17
CABG, n (%)	1	10 (9.2)		1 (2.9)	1	9 (12.0)	0.13
Operation*, n (%)	1	20 (18.3)		8 (23.5)	1	12 (16.0)	0.35
ICD/CRT, n (%)	1	6 (5.5)		1 (2.9)	1	5 (6.7)	0.43
Examinations							
TC, mg/dL	14	169 (153–201)	3	171 (152–192)	11	168 (156–206)	0.66
TG, mg/dL	17	121 (96–174)	3	117 (78–177)	14	133 (97–174)	0.59
LDL-C, mg/dL	18	98.2 ± 34.7	4	95.0 ± 32.8	14	99.8 ± 35.8	0.54
HDL-C, mg/dL	18	49.1 ± 14.2	4	48.3 ± 9.6	14	49.5 ± 16.1	0.65
BNP, pg/mL		81.7 (27.6–225)		84.4 (29.4–254)		80.9 (27.6–214)	0.88
LVEF, %	25	59.0 (44.6–67.9)	7	54.9 (40.1–65.5)	18	62.4 (45.3–68.5)	0.18
E/e'	48	11.5 (8.6–15.2)	16	13.0 (10.1–21.5)	32	10.3 (7.5–14.8)	0.07
TRPG, mm Hg	49	20.5 ± 7.5	17	19.3 ± 7.9	32	20.9 ± 7.4	0.07

Underlying CVD represents the indication to CR. P value showed the comparison between CR less than once a week group and CR once a week more group. *Operation indicates cardiac valvular operation. CR: cardiac rehabilitation; Missing: number of missing value; BMI: body mass index; HT: hypertension; DM: diabetes mellitus; DLP: dyslipidemia; CKD: chronic kidney disease; COPD: chronic obstructive pulmonary disease; CVD: cardiovascular disease; IHD: ischemic heart disease; HF: heart failure; MVD: macrovascular disease; RAAS: renin-angiotensin-aldosterone system; CCB: calcium channel blocker; BB: beta-blocker; MRA: mineral corticoid antagonist; PCI: percutaneous coronary intervention; CABG: coronary artery bypass graft; ICD: implantable cardioverter defibrillator; CRT: cardiac resynchronization therapy; TC: total cholesterol; TG: triglyceride; LDL-C: low-density lipoprotein cholesterol; HDL-C: high-density lipoprotein cholesterol; BNP: brain natriuretic peptide; LVEF: left ventricular ejection fraction; TRPG: tricuspid regurgitation pressure gradient.

### Changes in the results of the CPET by CR in the two groups

**Figure 2:**
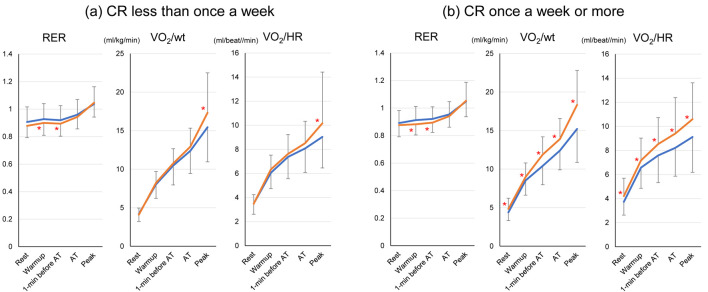
The changes in RER, VO_2_/wt, and VO_2_/HR in a cardiopulmonary exercise test from the initial test to a second test in patients who participated in CR less than once a week (a) and in those who participated in CR once a week or more (b) are shown. The blue line indicates the results at the initial test and the orange line indicates the results at the second test. *Indicates a significant difference between the initial and second tests. RER: respiratory exchange ratio; VO_2_/wt: oxygen uptake per body weight; VO_2_/HR: oxygen uptake per heart rate; AT: anaerobic threshold.

**Figure 3:**
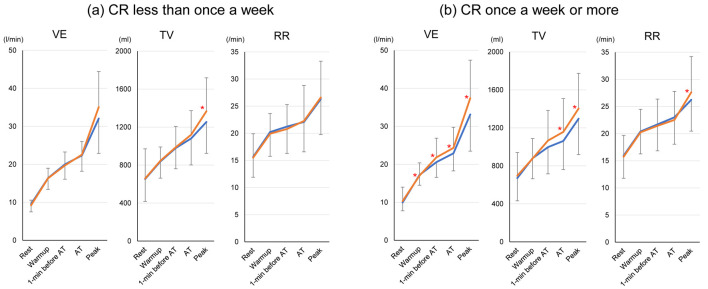
The changes in VE, TV, and RR in the cardiopulmonary exercise test from the initial test to the second test in patients who participated in CR less than once a week (a) and those who participated in CR once a week or more (b) are shown. The blue line indicates the results at the initial test and the orange line indicates the results at the second test. *Indicates a significant difference between the initial and second tests. VE: ventilation equivalent; TV: tidal volume; RR: respiratory rate; AT: anaerobic threshold.

**Figure 4:**
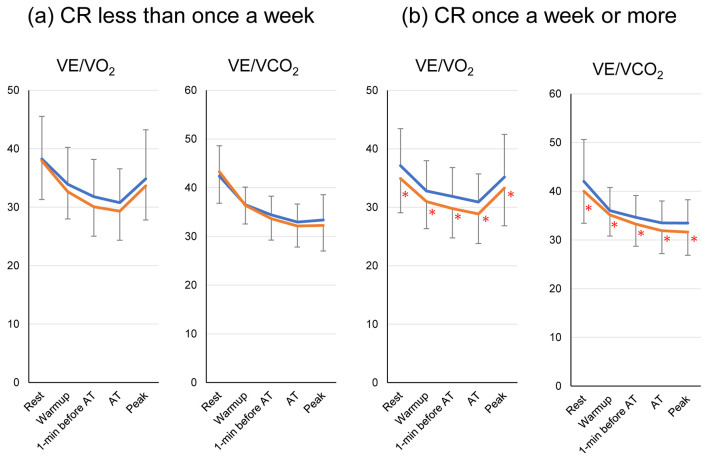
The changes in VE/VO_2_ and VE/VCO_2_ in the cardiopulmonary exercise test from the initial test to the second test in patients who participated in CR less than once a week (a) and those who participated in CR once a week or more (b) are shown. The blue line indicates the results at the initial test and the orange line indicates the results at the second test. *Indicates a significant difference between the initial and second tests. VE/VO_2_: ventilation equivalent per oxygen uptake; VE/VCO_2_; ventilation equivalent per carbon dioxide output; AT: anaerobic threshold.

**Figure 5:**
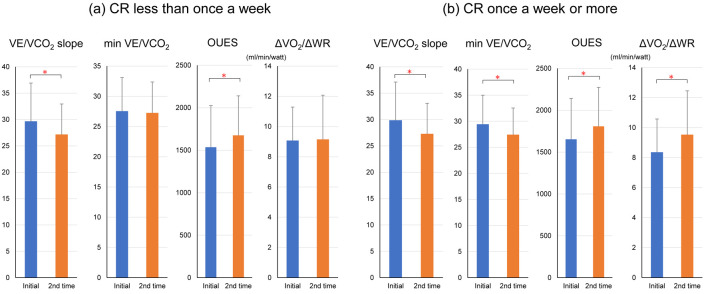
The changes in VE/VCO_2_ slope, minimum VE/VCO_2_, OUES, and ΔVO_2_/ΔWR in the cardiopulmonary exercise test from the initial test to the second test in patients who participated in CR less than once a week (a) and those who participated in CR once a week or more (b) are shown. *Indicates a significant difference between the initial and second tests. VE/VCO_2_: ventilation equivalent per carbon dioxide output; OUES: oxygen-uptake efficiency slope; ΔVO_2_/ΔWR: the ratio of the increase in oxygen uptake to the increase in work rate; AT: anaerobic threshold.

The changes in VO_2_ are shown in Figure 2. In patients who participated in CR less than once a week, VO_2_/wt and VO_2_/HR were significantly increased only at the peak period. In contrast, in patients who participated in CR once a week or more, these values were significantly increased from rest to peak periods. The changes in RER were similar in both groups. The changes regarding ventilatory equivalent are shown in Figure 3. In patients who participated in CR less than once a week, only tidal volume at the peak period significantly increased. In patients who participated in CR once a week or more, VE from warm-up to peak periods, and tidal volume from AT to peak periods, and RR at peak period were significantly increased. The changes in ventilatory efficiency are shown in Figure 4. While no significant changes were seen in patients who participated in CR less than once a week, those who participated in CR once a week or more group showed a significant decrease in VE/VO_2_ and VE/VCO_2_ from rest to peak periods. The major comparison of delta changes of individual parameters in CPET from the initial test to the second test between the two groups is shown in Table 2. In CR once a week or more group, delta VO_2_/wt and VO_2_/HR in 1-min before AT and AT periods significantly increased. In CR once a week or more group, delta VE in 1-min before AT period significantly improved. The changes in parameters by an interval analysis are shown in Figure 5. Even in patients who participated in CR less than once a week, the VE/VCO_2_ slope significantly decreased and OUES significantly increased. In patients who participated in CR once a week or more, minimum VE/VCO_2_ significantly decreased and ΔVO_2_/ΔWR significantly increased, in addition to significant changes in the VE/VCO_2_ slope and OUES. We performed sub-group analyses in ischemic heart disease and chronic heart failure groups. Those results also showed similar results to all patients (Supplementary Materials 1–8, cr.elmerpub.com).

**Table 2:** Major Differences in CPX From First to Second Test in Patients Who Participated in CR Once a Week or More Compared to Whom in CR Less Than Once a Week

	All cases (n = 110)	CR less than once a week (n = 35)	CR once a week more (n = 75)	P value
VO_2_/wt at 1-min before AT, mL/kg/min	1.30 (−0.30 to 2.20)	0.80 (−1.00 to 2.10)	1.50 (−0.10 to 2.60)	0.013
VO_2_/wt at AT, mL/kg/min	2.00 (0.40–4.00)	1.40 (−0.10 to 3.30)	2.30 (0.40–4.20)	0.035
VO_2_/HR at 1-min before AT, mL/beat/min	1.07 (0–1.76)	0.57 (−0.47 to 1.42)	1.23 (0.34–1.88)	0.007
VO_2_/HR at AT, mL/beat/min	0.92 (0.22–2.01)	0.50 (−0.47 to 1.67)	1.07 (0.34–2.26)	0.030
VE at 1-min before AT, L/min	0.40 (−0.70 to 3.10)	0 (−1.40 to 1.40)	0.90 (−0.50 to 3.50)	0.018
VE at AT, L/min	3.24 (0.05–7.19)	2.14 (−2.67 to 5.87)	4.84 (0.95–8.35)	0.066

CPX: cardiopulmonary exercise test; CR: cardiac rehabilitation; VO_2_/wt: oxygen uptake per body weight; VO_2_/HR: oxygen uptake per heart rate; AT: anaerobic threshold; VE: ventilation equivalent.

## Discussion

Outpatient CR once a week or more increased oxygen consumption and ventilator equivalent, and improved ventilator efficiency within 6 months. CR less than once a week also improved exercise tolerance, but the effects were small. While it would be better if patients participated in CR three times per week or more, as shown in the guidelines, even those who participated in a CR program once a week or more showed improved exercise tolerance. The cardiac rehabilitation dose around the world was investigated [15]. Cardiac rehabilitation programs were not enough in many countries. North America, south America, some Europe countries, and south Africa showed enough supervised cardiac rehabilitation dose. The other European countries, Asia, including Japan, Oceania, and Australia showed moderate number of supervised cardiac rehabilitation dose, but it is not sufficient. The frequency at which patients participate in outpatient CR in Japan is low. Kanaoka et al reported that patients participated in an average total of only nine (3–17) CR sessions over 180 days: only 3.1% of outpatients participated in CR two times per week or more and 17% discontinued CR within 1 month in patients with acute coronary syndrome who underwent percutaneous coronary intervention [16]. While it is desirable for patients to participate in outpatient exercise-based CR three times per week, it is also important to understand the effect of CR once a week or more for improving exercise tolerance in a real-world situation. In this study, the patients participated in outpatient CR on average 1.54 times per week. This value is relatively high compared to other Japanese data, since this study included only outpatients who continued CR and performed CPET two times: initially and within 6 months. Patients who participated in CR once a week or more showed a great increase in VO_2_/wt compared to those who participated in CR less than once a week. CR once a week or more should be effective for increasing exercise tolerance. With respect to patient backgrounds, only the use of diuretics differed between the groups. Although there were no significant differences, CR less than once a week group showed high rate of heart failure, high BNP, and low left ventricular ejection fraction. The high severity of heart failure might affect the high rate of diuretics usage. However, high severity of heart failure would not affect the poor improvement of exercise tolerance in CR less than once a week group. Because, subgroup analysis in heart failure patients also showed similar results to all patients. CR less than once a week also improved VO_2_/wt, but only the peak VO_2_/wt was increased. This is a good result in CR less than once a week, but VO_2_/wt at 1 min before AT and AT periods are important for cardiovascular patients. Those phases directly connect activities of daily living and quality of life for cardiovascular patients, because cardiovascular patients are limited with respect to their exercise intensity until AT to prevent the excessive activation of sympathetic nerve activity and metabolic acidosis, leading to arrhythmia, angina attack, and worsening heart failure [17]. The reasons for the improvement in VO_2_/wt are thought to be an increase in the blood’s oxygen transport capacity due to improved cardiopulmonary function and efficiency of oxygen utilization in muscles [18]. VO_2_/HR and ΔVO_2_/ΔWR are indicators of cardiac function [19]. The influence of beta-blockers on VO_2_/HR should also be considered [20]. This study revealed that CR once a week or more increased ΔVO_2_/ΔWR and VO_2_/HR under a 58.3% use rate of beta-blocker. This result reflects an increase in cardiac function by CR once a week or more, because this study used a pairwise analysis and there was almost no change in medications during the study period (data not shown). Patients who participated in CR once a week or more should be more likely to develop a habit. Periodic CR should be effective for increasing cardiac function. We could not assess the changes in left ventricular ejection fraction during the study period, due to the limited number of follow-up echocardiographic examinations. The improvements in ventilatory volume and ventilatory efficiency may have also contributed to the increase in VO_2_/wt. In this study, CR once a week or more increased VE, tidal volume, VE/VO_2_, VE/VCO_2_, and OUES. An increase in oxygen uptake directly influences exercise tolerance [21]. Respiratory muscles such as the diaphragm and intercostal muscles would be trained by periodic aerobic exercise [22]. In addition, CR increases parasympathetic activity and suppresses excessive sympathetic activation, which balances the autonomic nervous system and makes breathing more efficient during exercise [23]. These mechanisms are thought to have contributed to improvements in ventilation volume and ventilation efficiency. Exercise-based CR has been shown to improve heart failure [24]. VE/VCO_2_ slope and minimum VE/VCO_2_ are indicators of the severity of heart failure [19]. CR less than once a week improved VE/VCO_2_ slope, whereas CR once a week or more improved minimum VE/VCO_2_, in addition to VE/VCO_2_ slope. Periodic CR would be effective for improving heart failure. We could not assess changes in BNP during the study period, due to the limited number of follow-up laboratory examinations. High adherence to CR might support clinical awareness and new pharmacological treatment for cardiovascular disease and underlying disease, because it has been reported that CR program is associated with a very high low-density lipoprotein target achievement [25]. CR program also has a cardioprotective effects by suppressing inflammation and rescuing specific micro-RNAs and long non-coding RNAs modulated by cardiac stress [26]. Such a biological and epigenetic mechanism might affect our results.

### Limitations

This study has several limitations. This study does not have a control group. It has a possibility to contain selection bias, including the possibility that patients with higher participation frequency may have better baseline health status and functional capacity independent of CR participation. This is a retrospective observational single-center study and the sample size might affect the results. To confirm the effects of CR once a week or more, further studies will be needed. We could not investigate the changes without CPET during the study period, due to the limited number of follow-up examinations.

### Conclusion

Even a CR program once a week or more was effective for improving exercise tolerance. The improvements of CPET in patients who participated CR once a week or more were acceptable.

## Supplementary Material

Suppl 1 Changes in RER, VO_2_/wt, and VO_2_/HR in a cardiopulmonary exercise test from the initial test to a second test in patients with ischemic heart disease who participated in CR less than once a week and in those who participated in CR once a week or more.

Suppl 2 Changes in ventilatory volume in a cardiopulmonary exercise test from the initial test to a second test in patients with ischemic heart disease who participated in CR less than once a week group and those who participated in CR once a week or more.

Suppl 3 Changes in ventilatory efficiency in the cardiopulmonary exercise test from the initial test to the second test in patients with ischemic heart disease who participated in CR less than once a week and those who participated in CR once a week or more.

Suppl 4 Changes by interval analyses in a cardiopulmonary exercise test from the initial test to the second test in patients with ischemic heart disease who participated in CR less than once a week and those who participated in CR once a week or more.

Suppl 5 Changes in RER, VO_2_/wt, and VO_2_/HR in a cardiopulmonary exercise test from the initial test to a second test in patients with chronic heart failure who participated in CR less than once a week and in those who participated in CR once a week or more.

Suppl 6 Changes in ventilatory volume in a cardiopulmonary exercise test from the initial test to a second test in patients with chronic heart failure who participated in CR less than once a week group and those who participated in CR once a week or more.

Suppl 7 Changes in ventilatory efficiency in the cardiopulmonary exercise test from the initial test to the second test in patients with chronic heart failure who participated in CR less than once a week and those who participated in CR once a week or more.

Suppl 8 Changes by interval analyses in a cardiopulmonary exercise test from the initial test to the second test in patients with chronic heart failure who participated in CR less than once a week and those who participated in CR once a week or more.

## Data Availability

The data supporting the findings of this study are available from the corresponding author upon reasonable request.

## References

[1] Makita S, Yasu T, Akashi YJ, Adachi H, Izawa H, Ishihara S, Iso Y (2022). JCS/JACR 2021 guideline on rehabilitation in patients with cardiovascular disease. Circ J.

[2] Anderson L, Oldridge N, Thompson DR, Zwisler AD, Rees K, Martin N, Taylor RS (2016). Exercise-based cardiac rehabilitation for coronary heart disease: cochrane systematic review and meta-analysis. J Am Coll Cardiol.

[3] O'Connor CM, Whellan DJ, Lee KL, Keteyian SJ, Cooper LS, Ellis SJ, Leifer ES (2009). Efficacy and safety of exercise training in patients with chronic heart failure: HF-ACTION randomized controlled trial. JAMA.

[4] Mandic S, Stevens E, Hodge C, Brown C, Walker R, Body D, Barclay L (2016). Long-term effects of cardiac rehabilitation in elderly individuals with stable coronary artery disease. Disabil Rehabil.

[5] Caminiti G, Volterrani M, Iellamo F, Marazzi G, Silvestrini M, Giamundo DM, Morsella V (2025). Exercise training for patients with heart failure and preserved ejection fraction. A narrative review. Monaldi Arch Chest Dis.

[6] Adachi H (2017). Cardiopulmonary exercise test. Int Heart J.

[7] Wasserman K, Stringer WW, Casaburi R, Koike A, Cooper CB (1994). Determination of the anaerobic threshold by gas exchange: biochemical considerations, methodology and physiological effects. Z Kardiol.

[8] Ponikowski P, Voors AA, Anker SD, Bueno H, Cleland JGF, Coats AJS, Falk V (2016). 2016 ESC Guidelines for the diagnosis and treatment of acute and chronic heart failure: The Task Force for the diagnosis and treatment of acute and chronic heart failure of the European Society of Cardiology (ESC)Developed with the special contribution of the Heart Failure Association (HFA) of the ESC. Eur Heart J.

[9] Yancy CW, Jessup M, Bozkurt B, Butler J, Casey DE, Colvin MM, Drazner MH (2017). 2017 ACC/AHA/HFSA Focused Update of the 2013 ACCF/AHA Guideline for the Management of Heart Failure: A Report of the American College of Cardiology/American Heart Association Task Force on Clinical Practice Guidelines and the Heart Failure Society of America. Circulation.

[10] Dibben GO, Faulkner J, Oldridge N, Rees K, Thompson DR, Zwisler AD, Taylor RS (2023). Exercise-based cardiac rehabilitation for coronary heart disease: a meta-analysis. Eur Heart J.

[11] Brown TM, Pack QR, Aberegg E, Brewer LC, Ford YR, Forman DE, Gathright EC (2024). Core components of cardiac rehabilitation programs: 2024 update: a scientific statement from the American Heart Association and the American Association of Cardiovascular and Pulmonary Rehabilitation. Circulation.

[12] Visseren FLJ, Mach F, Smulders YM, Carballo D, Koskinas KC, Back M, Benetos A (2021). 2021 ESC Guidelines on cardiovascular disease prevention in clinical practice. Eur Heart J.

[13] Inada Y, Suematsu Y, Matsuda T, Yano Y, Morita K, Bando K, Teshima R (2024). Effect of left ventricular diastolic dysfunction on the cardiopulmonary exercise test in patients with cardiovascular disease. Am J Cardiol.

[14] Yano Y, Suematsu Y, Matsuda T, Tsukahara K, Shirosaki M, Matsuo S, Fujimi K (2024). Usefulness of the cardiopulmonary exercise test up to the anaerobic threshold for pati-ents aged >/= 80 years with cardiovascular disease on cardiac rehabilitation. J Rehabil Med.

[15] Chaves G, Turk-Adawi K, Supervia M, Santiago de Araujo Pio C, Abu-Jeish AH, Mamataz T, Tarima S (2020). Cardiac rehabilitation dose around the world: variation and correlates. Circ Cardiovasc Qual Outcomes.

[16] Kanaoka K, Iwanaga Y, Nakai M, Nishioka Y, Myojin T, Kubo S, Okada K (2022). Outpatient cardiac rehabilitation dose after acute coronary syndrome in a nationwide cohort. Heart.

[17] Fuchs AR, Meneghelo RS, Stefanini E, De Paola AV, Smanio PE, Mastrocolla LE, Ferraz AS (2009). Exercise may cause myocardial ischemia at the anaerobic threshold in cardiac rehabilitation programs. Braz J Med Biol Res.

[18] Murata M, Adachi H, Oshima S, Kurabayashi M (2017). Influence of stroke volume and exercise tolerance on peak oxygen pulse in patients with and without beta-adrenergic receptor blockers in patients with heart disease. J Cardiol.

[19] Tran D (2018). Cardiopulmonary exercise testing. Methods Mol Biol.

[20] Taylor JL, Bonikowske AR, Olson TP (2021). Optimizing outcomes in cardiac rehabilitation: the importance of exercise intensity. Front Cardiovasc Med.

[21] Albouaini K, Egred M, Alahmar A, Wright DJ (2007). Cardiopulmonary exercise testing and its application. Postgrad Med J.

[22] Powers SK, Coombes J, Demirel H (1997). Exercise training-induced changes in respiratory muscles. Sports Med.

[23] Meyer K (2001). Exercise training in heart failure: recommendations based on current research. Med Sci Sports Exerc.

[24] Bozkurt B, Fonarow GC, Goldberg LR, Guglin M, Josephson RA, Forman DE, Lin G (2021). Cardiac rehabilitation for patients with heart failure: JACC expert panel. J Am Coll Cardiol.

[25] Maloberti A, Diri BP, Bellomare M, Tognola C, Shkodra A, Algeri M, Prencipe GP (2026). Low density lipoprotein target achivement in very high and extreme cardiovascular risk patients during a cardiac rehabilitation program. Nutr Metab Cardiovasc Dis.

[26] Visco V, Forte M, Giallauria F, D'Ambrosio L, Piccoli M, Schiattarella GG, Mancusi C (2025). Epigenetic mechanisms underlying the beneficial effects of cardiac rehabilitation. An overview from the working groups of "cellular and molecular biology of the heart" and "cardiac rehabilitation and cardiovascular prevention" of the Italian Society of Cardiology (SIC). Int J Cardiol.

