# Supplementary material for: Strong Adherence to Cardiac Rehabilitation Program Improves Exercise Tolerance in Outpatients With Cardiovascular Disease
Source: Cardiol Res. 2026 Jun 5;17(3):181–9. doi: 10.14740/cr2213 (PMC13278718; doi:10.14740/cr2213)

**Suppl 2. Changes in ventilatory volume in a cardiopulmonary exercise test from the initial test to a 2^nd^ test in patients with ischemic heart disease who participated in CR less than once a week group and those who participated in CR once a week or more**

The changes in VE, TV, and RR in the cardiopulmonary exercise test from the initial test to the 2^nd^ test in patients with ischemic heart disease who participated in CR less than once a week (a) and those who participated in CR once a week or more (b) are shown. The orange line indicates the results in the initial test and the blue line indicates the results in the 2^nd^ test. * Indicates a significant difference between the initial and 2^nd^ tests. VE; ventilation equivalent, TV; tidal volume, RR; respiratory rate, AT; anaerobic threshold.


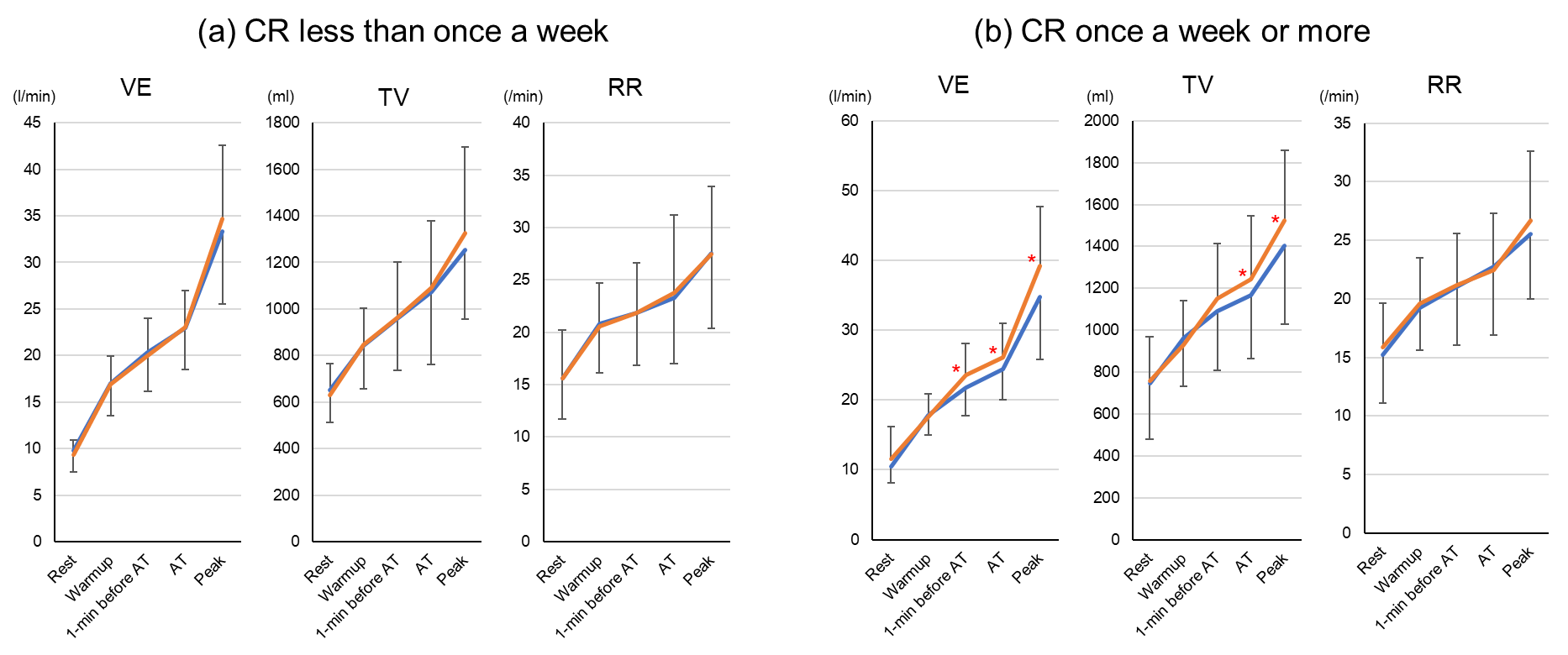

Supplement: Suppl 2 — Changes in ventilatory volume in a cardiopulmonary exercise test from the initial test to a second test in patients with ischemic heart disease who participated in CR less than once a week group and those who participated in CR once a week or more. [file cr-17-03-181-s002.docx]
