# Supplementary material for: Strong Adherence to Cardiac Rehabilitation Program Improves Exercise Tolerance in Outpatients With Cardiovascular Disease
Source: Cardiol Res. 2026 Jun 5;17(3):181–9. doi: 10.14740/cr2213 (PMC13278718; doi:10.14740/cr2213)

**Suppl 3. Changes in ventilatory efficiency in the cardiopulmonary exercise test from the initial test to the 2^nd^ test in patients with ischemic heart disease who participated in CR less than once a week and those who participated in CR once a week or more**

The changes in VE/VO_2_ and VE/VCO_2_ in the cardiopulmonary exercise test from the initial test to the 2^nd^ test in patients with ischemic heart disease who participated in CR less than once a week (a) and those who participated in CR once a week or more (b) are shown. The orange line indicates the results in the initial test and the blue line indicates the results in the 2^nd^ test. * Indicates a significant difference between the initial and 2^nd^ tests VE/VO_2_; ventilation equivalent per oxygen uptake, VE/VCO_2_; ventilation equivalent per carbon dioxide output, AT; anaerobic threshold.


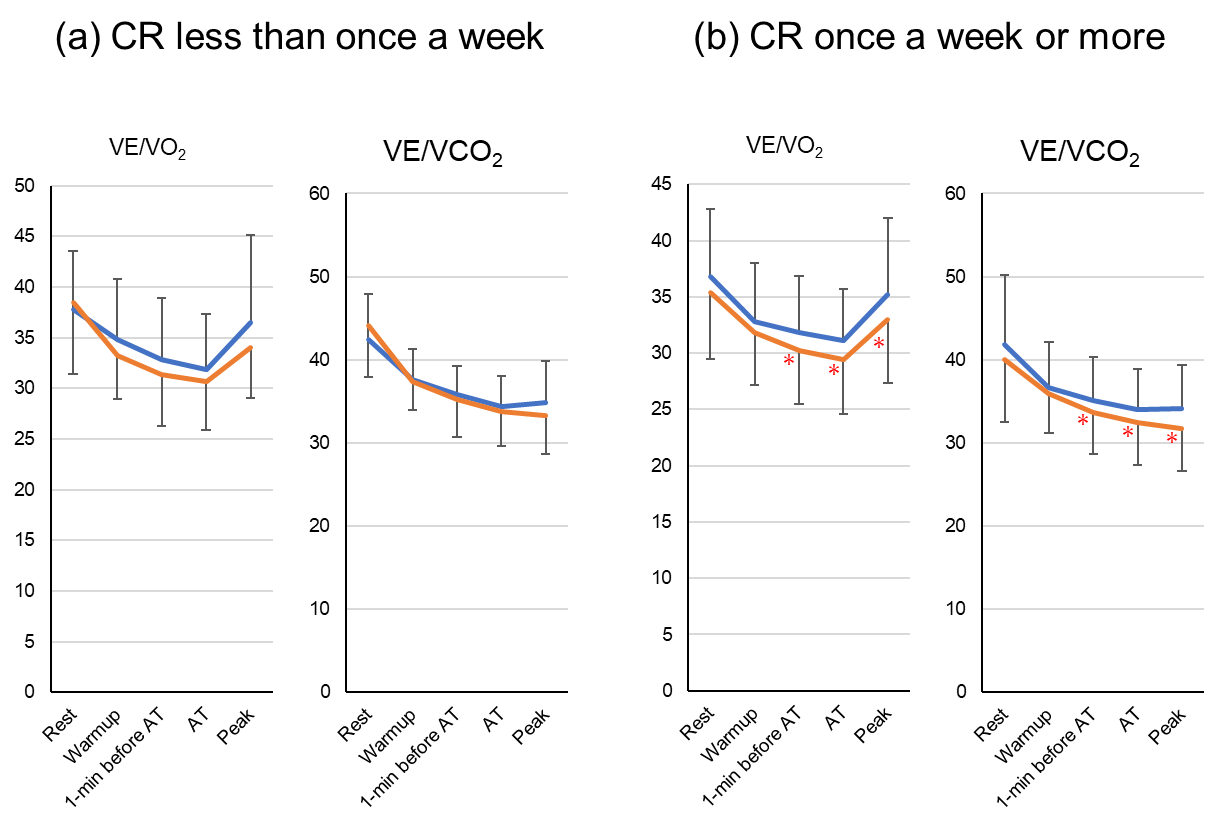

Supplement: Suppl 3 — Changes in ventilatory efficiency in the cardiopulmonary exercise test from the initial test to the second test in patients with ischemic heart disease who participated in CR less than once a week and those who participated in CR once a week or more. [file cr-17-03-181-s003.docx]
