# Supplementary material for: Strong Adherence to Cardiac Rehabilitation Program Improves Exercise Tolerance in Outpatients With Cardiovascular Disease
Source: Cardiol Res. 2026 Jun 5;17(3):181–9. doi: 10.14740/cr2213 (PMC13278718; doi:10.14740/cr2213)

**Suppl 5. Changes in RER, VO_2_/wt, and VO_2_/HR in a cardiopulmonary exercise test from the initial test to a 2^nd^ test in patients with chronic heart failure who participated in CR less than once a week and in those who participated in CR once a week or more**

The changes in RER, VO_2_/wt, and VO_2_/HR in a cardiopulmonary exercise test from the initial test to a 2^nd^ test in patients with chronic heart failure who participated in CR less than once a week (a) and in those who participated in CR once a week or more (b) are shown. The orange line indicates the results at the initial test and the blue line indicates the results at the 2^nd^ test. * Indicates a significant difference between the initial and 2^nd^ tests. RER; respiratory exchange ratio, VO_2_/wt; oxygen uptake per body weight, VO_2_/HR; oxygen uptake per heart rate, AT; anaerobic threshold.


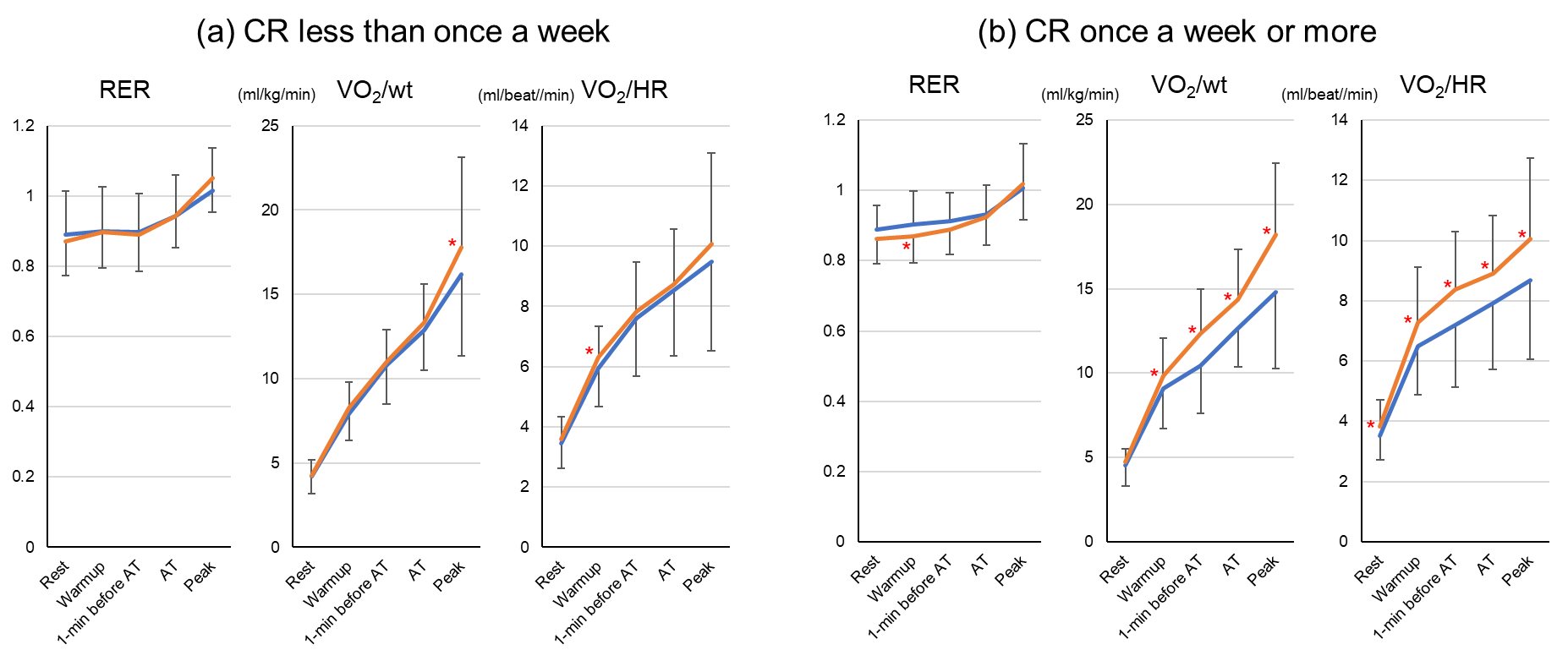

Supplement: Suppl 5 — Changes in RER, VO2/wt, and VO2/HR in a cardiopulmonary exercise test from the initial test to a second test in patients with chronic heart failure who participated in CR less than once a week and in those who participated in CR once a week or more. [file cr-17-03-181-s005.docx]
