# Supplementary material for: Strong Adherence to Cardiac Rehabilitation Program Improves Exercise Tolerance in Outpatients With Cardiovascular Disease
Source: Cardiol Res. 2026 Jun 5;17(3):181–9. doi: 10.14740/cr2213 (PMC13278718; doi:10.14740/cr2213)

**Suppl 8. Changes by interval analyses in a cardiopulmonary exercise test from the initial test to the 2^nd^ test in patients with chronic heart failure who participated in CR less than once a week and those who participated in CR once a week or more**

The changes in VE/VCO_2_ slope, minimum VE/VCO_2_, OUES, and ΔVO_2_/ΔWR in the cardiopulmonary exercise test from the initial test to the 2^nd^ test in patients with chronic heart failure who participated in CR less than once a week (a) and those who participated in CR once a week or more (b) are shown. * Indicates a significant difference between the initial and 2^nd^ tests. VE/VCO_2_; ventilation equivalent per carbon dioxide output, OUES; oxygen-uptake efficiency slope, ΔVO_2_/ΔWR; the ratio of the increase in oxygen uptake to the increase in work rate, AT; anaerobic threshold.


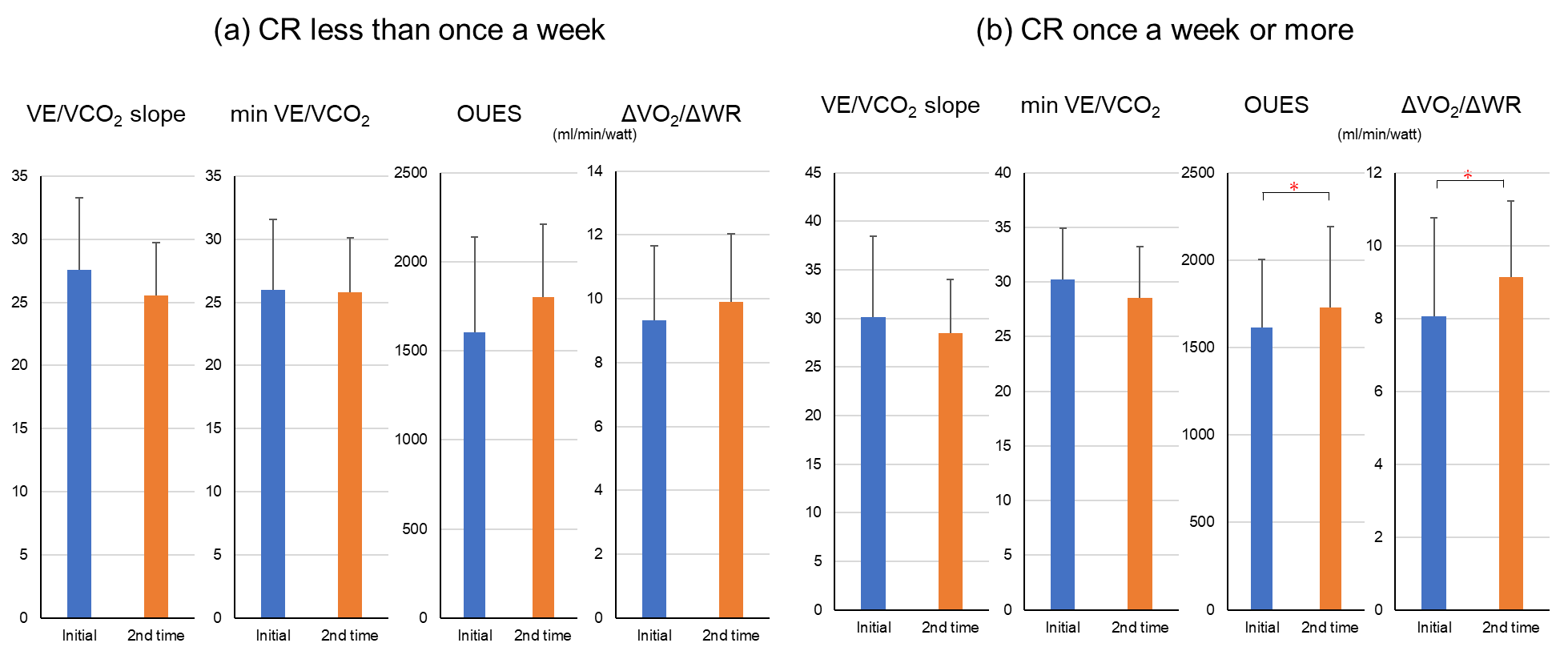

Supplement: Suppl 8 — Changes by interval analyses in a cardiopulmonary exercise test from the initial test to the second test in patients with chronic heart failure who participated in CR less than once a week and those who participated in CR once a week or more. [file cr-17-03-181-s008.docx]
